# Catechol-O-Methyltransferase Val158Met Polymorphism Modulates Gray Matter Volume and Functional Connectivity of the Default Mode Network

**DOI:** 10.1371/journal.pone.0078697

**Published:** 2013-10-16

**Authors:** Tian Tian, Wen Qin, Bing Liu, Dawei Wang, Junping Wang, Tianzi Jiang, Chunshui Yu

**Affiliations:** 1 Department of Radiology and Tianjin Key Laboratory of Functional Imaging, Tianjin Medical University General Hospital, Tianjin, China; 2 Brainnetome Center, Institute of Automation, Chinese Academy of Sciences, Beijing, China; 3 National Laboratory of Pattern Recognition, Institute of Automation, Chinese Academy of Sciences, Beijing, China; 4 Key Laboratory for NeuroInformation of Ministry of Education, School of Life Science and Technology, University of Electronic Science and Technology of China, Chengdu, China; 5 The Queensland Brain Institute, the University of Queensland, Brisbane, Australia; University of Pennsylvania, United States of America

## Abstract

The effect of catechol-O-methyltransferase (COMT) Val158Met polymorphism on brain structure and function has been previously investigated separately and regionally; this prevents us from obtaining a full picture of the effect of this gene variant. Additionally, gender difference must not be overlooked because estrogen exerts an interfering effect on COMT activity. We examined 323 young healthy Chinese Han subjects and analyzed the gray matter volume (GMV) differences between Val/Val individuals and Met carriers in a voxel-wise manner throughout the whole brain. We were interested in genotype effects and genotype × gender interactions. We then extracted these brain regions with GMV differences as seeds to compute resting-state functional connectivity (rsFC) with the rest of the brain; we also tested the genotypic differences and gender interactions in the rsFCs. Val/Val individuals showed decreased GMV in the posterior cingulate cortex (PCC) compared with Met carriers; decreased GMV in the medial superior frontal gyrus (mSFG) was found only in male Val/Val subjects. The rsFC analysis revealed that both the PCC and mSFG were functionally correlated with brain regions of the default mode network (DMN). Both of these regions showed decreased rsFCs with different parts of the frontopolar cortex of the DMN in Val/Val individuals than Met carriers. Our findings suggest that the COMT Val158Met polymorphism modulates both the structure and functional connectivity within the DMN and that gender interactions should be considered in studies of the effect of this genetic variant, especially those involving prefrontal morphology.

## Introduction

Catechol-O-methyltransferase (COMT) catalyzes the degradation of synaptic dopamine (DA) in the brain, especially in the prefrontal cortex (PFC), where COMT may account for more than half of DA decline because of the lack of DA transporter in PFC synapses [1,2]. The COMT gene is located on chromosome 22q11 and contains a functional polymorphism (Val158Met) that results in a fourfold decrease in enzymatic activity at body temperature in Met-allele carriers [1]. Decreased enzymatic activity leads to increased synaptic DA concentrations, which may affect cognitive and emotional functions via modulation of brain structure and function, especially in the PFC [3-9]. However, dopaminergic modulation of phenotypes is complex and has been described as an inverted U-shaped relationship [10,11] in which both the lowest and highest DA levels may impair behavioral performance [12-15]. 

 Although Met allele carriers exhibit better performance in episodic memory and executive functions [16-26], Val carriers show increased activation of the prefrontal cortex during a variety of cognitive tasks [17,21,27,28]. In contrast, Val carriers exhibit better performance in emotion processing tasks [29,30]. These individuals show greater activation during emotional awareness [30] and regulation tasks [31]; however, they exhibit decreased limbic and prefrontal reactivity [32,33] and prefrontal-limbic connectivity [32,34] while processing unpleasant stimuli. These inverse effects have been ascribed to selective modulation of COMT Val158Met on prefrontal dopamine-associated processing and opposing effects of the Val158Met genotype on stable and flexible demands of cognition [35,36]. The effects of COMT Val158Met on brain function have also been investigated during non-task states. Resting-state electroencephalogram has revealed that Val/Val individuals exhibit lower baseline prefrontal activation [37] but greater connectivity strengths between frontal and temporal/parietal areas [38]. Moreover, previous resting-state fMRI studies have reported that Val/Val individuals showed weaker prefrontal-related resting-state functional connectivity (rsFC) in the default mode network (DMN) [39] and greater prefrontal-related rsFC in the executive control network than Met carriers [40]. Beyond the modulation of COMT Val158Met on functional brain characteristics, the Val158Met polymorphism has been shown to affect structural profiles of the brain. Most structural MRI studies have suggested that the COMT Val158Met was related to structural differences in healthy people [4,41-50]; however, one recent study reported no difference in gray matter volume (GMV) between genotypes in healthy young adults [51]. 

As mentioned above, most previous studies performed either structural or functional analysis, but to date, these methods have not been combined to provide a complete picture of the effect of COMT Val158Met polymorphism on the brain in healthy individuals. Only one study of healthy young men has combined GMV and rsFC analyses; these authors reported that the COMT Val158Met polymorphism affected the rsFC of the PFC, although this difference occurred in the absence of any alterations in gray matter [40]. Another study of healthy children has combined GMV and activation analyses; these authors reported increased GMV in the left hippocampal head and increased activation in the right parahippocampal gyrus during emotional processing in Met carriers [52]. Inspired by these multimodal imaging studies aiming at the structure-functional interactions [53], we will explore whether the rsFC differences between genotypes are driven by the corresponding structural differences. Additionally, most previous studies have followed a hypothesis-driven method and focused on regional changes, especially in the PFC and hippocampus. In the present study, we combined voxel-based structural (GMV) and rsFC analyses throughout the whole brain to characterize the structural and functional differences between Val158Met genotypes. 

Estrogen can down-regulate COMT activity [54,55], resulting in decreased COMT activity in females compared with that in males. After Gogos and colleagues demonstrated the gender effects on DA levels and behaviors in COMT knockout mice [56], gender differences in the effects of COMT Val158Met on structural brain characteristics [50] and behavioral performance [23,57-62] have also been identified in healthy subjects. However, it should be noted that several previous studies have reported that there are no gender differences in brain structure and behavioral performance [51,63]. As a potential interacting factor, gender differences must not be ignored when considering the impact of COMT Val158Met on the human brain. 

In the present study, we used a voxel-based morphometry (VBM) technique to investigate both the genotypic effects and gender interactions on GMV throughout the whole brain. We then extracted brain regions with GMV differences as seed regions to compute rsFC with the rest of the brain; we also tested group differences and gender interactions in the rsFCs. Notably, we only recruited healthy young adults aged 18-31 years because previous studies have reported the interaction between COMT Val158Met genotype and age on brain structure [64] and functions [20,65,66]. 

## Materials and Methods

### Subjects

A total of 323 healthy young right-handed subjects (mean age: 22.7 ± 2.5 years; 157 males) participated in this study. Participants were carefully screened to ensure that there was no history of psychiatric or neurological illness, psychiatric treatment, or drug or alcohol abuse, and had no contraindications for MRI examinations. Only Chinese Han subjects were included to avoid artifacts of population stratification. All subjects were strongly right-handed according to the Chinese edition of the Edinburgh Handedness Inventory [67]. After a complete description of the study, all subjects provided written informed consent. The protocol was approved by the Ethics Committee of Tianjin Medical University. Subject intelligence quotient (IQ) scores were measured using the Chinese Revised Wechsler Adult Intelligence Scale (WAIS-RC) [68]. Depression scores were evaluated with the Beck Depression Inventory (BDI) [69]. Anxiety levels were obtained using the Self-Rating Anxiety Scale (SAS) [70]. The Tridimensional Personality Questionnaire (TPQ) was used to quantify each temperamental characteristic [71]. The above behavioral scales are known to reflect structural and functional characteristics of the brain [45,58,72-74] and were compared between genotypes. All data are stored according to the requirements of our hospital at the Tianjin Key Laboratory of Functional Imaging, where the experiments were done. These data are not publicly deposited.

### Genotyping

We extracted genomic DNA from 3000 µl of whole blood using the EZgeneTM Blood gDNA Miniprep Kit (Biomiga Inc, San Diego, CA, USA). We then genotyped the COMT rs4680 in each subject using the PCR and ligation detection reaction (LDR) method [75,76] with technical support from the Shanghai Biowing Applied Biotechnology Company. The PCR primer sequences of COMT were as follows: forward: 5’ GGGCCTACTGTGGCTACTCA 3’, and reverse: 5’ CCCTTTTTCCAGGTCTGACA 3’. PCR was performed at a 20 μL volume containing 1 μL genomic DNA, 0.4 μL primer mixture, 2 μL dNTPs, 0.6 μL Mg^2+^, 2 μL buffer, 4 μL Q-Solution, and 0.3 μL Taq DNA polymerase. The amplification protocol consisted of an initial denaturation and enzyme activation phase at 95°C for 15 min, followed by 35 cycles of denaturation at 94°C for 30 sec, annealing at 59°C for COMT rs4680 for 1 min and 30 sec, extension at 72°C for 1 min, and then a final extension at 72°C for 7 min. PCR products were verified in 3% agarose gels that had been stained with ethidium bromide to regulate the amount of DNA added to the LDR. 

Three probes were designed for the LDR reactions for each SNP, including one common probe (rs4680_modify: P-GCCAGCGAAATCCACCATCCGCTGGTTTTTTTTTTTTTTTTTTTT-FAM) and two discriminating probes for the two alleles of the SNP (rs4680_A: TTTTTTTTTTTTTTTTTTTTCAGGCATGCACACCTTGTCCTTCAT; and rs4680_G: TTTTTTTTTTTTTTTTTTTTTTCAGGCATGCACACCTTGTCCTTCAC). These reactions were carried out in a 10 μL mixture containing 1 μL buffer, 1 μL probe mix, 0.05 μL Taq DNA ligase, 1 μL PCR product, and 6.95 μL deionized water. The reaction program consisted of initial heating at 95°C for 2 min, followed by 35 cycles of 30 sec at 94°C and 2 min at 50°C. Reactions were stopped by chilling the tubes in an ethanol–dry ice bath and adding 0.5 mL of 0.5 mM EDTA. Aliquots of 1 μL of the reaction products were mixed with 1 μL of loading buffer (83% formamide, 8.3 mM EDTA and 0.17% blue dextran) and 1 μL ABI GS-500 Rox-Fluorescent molecular weight marker, which were denatured at 95°C for 2 min and chilled rapidly on ice prior to being loaded on a 5 Murea-5% polyacrylamide gel and electrophoresed on an ABI 3100 DNA sequencer at 3000 V. Finally, fluorescent ligation products were analyzed and quantified using the ABI GeneMapper software. 

Twenty-one of the 323 subjects were excluded from further analysis due to genotyping failure. The COMT rs4680 genotype distribution in the sample was in Hardy–Weinberg equilibrium (*P* > 0.05). The frequencies of the COMT genotypes are presented in Table 1. No genotype distribution differences were found between males and females. Subjects who were homozygous and heterozygous for the A-allele were merged into a group of A-allele carriers and compared with homozygotes for the G-allele; this method has been used previously to address skewed genotypic distributions [45,48,77,78].

**Table 1:** The number of samples collected, gender ratio and distributions of COMT Val158Met polymorphism.

Gender	N (%)	COMT genotypes(%)			Allele (%)	
		Val/Val	Val/Met	Met/Met	Val	Met
Male	144 (47.68)	64 (44.44)	62 (43.06)	18 (12.50)	190 (65.97)	98 (34.03)
Female	158 (52.32)	77 (48.73)	69 (43.67)	12 (7.59)	223 (70.57)	93 (29.43)
Total	302 (100.00)	141 (46.69)	131 (43.38)	30 (9.93)	413 (68.38)	191 (31.62)

### Image acquisition

MR images were acquired using a Signa HDx 3.0 Tesla MR scanner (General Electric, Milwaukee, WI, USA). Tight but comfortable foam padding was used to minimize head motion and earplugs were used to reduce scanner noise. Resting-state functional MRI data were obtained using the Single-Shot Echo-Planar Imaging sequence (SS-EPI) with the following imaging parameters: repetition time (TR)/echo time (TE) = 2000/30 ms; field of view (FOV) = 240 mm × 240 mm; matrix = 64 × 64; flip angle (FA) = 90°, slice thickness = 4 mm; no gap; 40 transversal slices; 180 volumes. During fMRI scans, all subjects were instructed to keep their eyes closed, to stay as motionless as possible, to think of nothing in particular, and not to fall asleep. Sagittal 3D T1-weighted images were acquired by a brain volume (BRAVO) sequence (TR/TE = 8.1/3.1 ms; inversion time = 450 ms; FA = 13°; FOV = 256 mm × 256 mm; matrix = 256 × 256; slice thickness = 1 mm, no gap; 176 sagittal slices). 

### Data preprocessing

All structural images were carefully checked slice by slice. Three of the remaining 302 subjects were excluded due to poor image quality or visible structural abnormality. Thus, a total of 299 subjects were included in the VBM analysis. The structural MR images were segmented into gray matter (GM), white matter and cerebrospinal fluid (CSF) using the new segmentation model in the Statistical Parametric Mapping software package (SPM8, http://www.fil.ion.ucl.ac.uk/spm). The new segmentation model is an extension of the “unified segmentation” algorithm [79], which includes additional tissue probability maps to better model CSF and other non-brain voxels, resulting in a more accurate segmentation. Following segmentation, GM population templates were generated from the entire image dataset using diffeomorphic anatomical registration through the exponentiated Lie algebra (DARTEL) technique [80]. After an initial affine registration of the GM DARTEL template to the tissue probability map in the Montreal Neurological Institute (MNI) space (http://www.mni.mcgill.ca/), non-linear warping of GM images was performed to the DARTEL GM template in the MNI space with a resolution of 1.5-mm^3^ (as recommended for the DARTEL procedure). The GMV of each voxel was obtained by multiplying the GM concentration map by the non-linear determinants derived during spatial normalization. Finally, to compensate for residual between-subjects anatomical differences, the GMV images were smoothed with a full width at half maximum (FWHM) kernel of 8 mm. In effect, the regional GMV represents normalized GMVs after removing the confounding effects of variance in individual brain sizes. After spatial pre-processing, normalized, modulated, and smoothed GMV maps were used for statistical analysis. 

Each of the functional MR images from the 299 subjects was carefully checked slice by slice to exclude scans with obvious distortions, signal loss, or artifacts. Functional MRI data for 299 subjects were preprocessed using the Data Processing Assistant for Resting-State fMRI (DPARSF) [81]. The first 10 volumes of each subject were discarded for signal equilibrium and participants’ adaptation to scanning noise. The remaining 170 volumes were first corrected for the acquisition time delay between slices. Head motion parameters were estimated and each volume was realigned to the mean whole volume map to correct for geometrical displacements using a six-parameter rigid-body transformation. Eleven participants were excluded from further analysis because their maximum displacement in any of the orthogonal directions (x, y, z) more than 2 mm, or there was a maximum rotation (x, y, z) greater than 2.0°. Thus, a total of 288 subjects were included in further rsFC preprocessing. We also calculated framewise displacement (FD), which indexes volume-to-volume changes in head position. These changes were obtained from derivatives of the rigid body realignment estimates that are used to realign blood oxygen level-dependent (BOLD) data during fMRI preprocessing [82,83]. There were no main effect of the genotype and interaction effect between genotype and gender on the FD (Table S1). Then all data were spatially normalized to the standard echo-planar imaging (EPI) template, and resampled to 3-mm^3^ voxels. The normalized data were smoothed with an 8-mm FWHM. Linear drifts were removed, and a temporal filter (0.01-0.08 Hz) was performed to reduce the effect of low-frequency drifts and high-frequency noise. Finally, a multiple regression method was performed to remove the possible influences of confounding factors, including six estimated motion parameters and average BOLD signals in the whole brain and CSF and white matter regions. The whole brain, CSF and white matter masks were defined using the tissue probabilistic maps in MNI space [81], i.e., the whole brain mask was thresholded at 50%, the white matter mask was thresholded at 90%, and the CSF mask was thresholded at 70%.

### Statistical analysis

Statistical analyses for the demographic, cognitive and psychological data were performed using Statistical Package for the Social Sciences version 18.0 (SPSS, Chicago, IL, USA) for Windows. A two-way (genotype and gender) analysis of variance (ANOVA) was used to evaluate the main effects of genotype and gender and their interactions on age, years of education, IQ, depression scores, anxiety levels and personality traits. 

The voxel-based comparisons of GMV were performed using a factorial (genotype by gender) ANOVA (*P* < 0.001, cluster size > 200 voxels). Brain regions with significant genotypic effect and genotype × gender interaction in GMV were extracted and defined as seed regions for the rsFC analysis. For each subject, the correlation coefficient between the mean time series of each seed region and that of each voxel in the whole brain was computed and transformed into a z-value to improve normality. Subsequently, individuals’ z-values were entered into a random effects one-sample t-test to identify brain regions exhibiting significant positive correlations with the seed region. Significant rsFC maps were corrected for multiple comparisons using the Family Wise Error (FWE, *P* < 0.05) method. A mask of brain areas with significant positive rsFCs with the seed region was generated and applied in a two-way (genotype and gender) ANOVA. Correction for multiple comparisons was performed using the Monte Carlo simulation with a corrected threshold of *P* < 0.05 and a cluster size of at least 11 voxels (AlphaSim program. Parameters: single voxel *P* = 0.001, FWHM = 8 mm, cluster connection radius r = 5 mm; with a GM mask and a resolution of 3-mm^3^). 

To remove potential influence by age, years of education, IQ, depression scores, anxiety levels and personality traits on the GMV and rsFC analyses, we extracted brain regions with significant differences in GMV and rsFCs; and repeated the statistical analyses using these measures as covariates of no interest. To explore whether rsFC differences are driven by the corresponding structural differences, we repeated the rsFC analyses using the GMVs of the regions of interest (ROIs) as covariates of no interest to remove potential variations in rsFCs related to GMV [53]. 

The main effects of genotype and gender, and the genotype × gender interaction were reported. If the interaction was significant, *post-hoc* comparisons were performed to determine genotypic differences in female and male subjects, respectively. 

## Results

### Demographic and genetic characteristics

The VBM analysis included 299 subjects, which consisted of 139 Val/Val, 130 Val/Met, and 30 Met/Met individuals (Table 2). The rsFC analysis included 288 subjects, which consisted of 137 Val/Val, 124 Val/Met, and 27 Met/Met individuals (Table 2). Because of the relative low frequency of Met homozygotes (4-5 times lower than Val homozygotes), we merged the Met homozygotes and Met heterozygotes into a group of Met carriers. Detailed demographic, cognitive and psychological data are summarized in Table S2, Table S3 and Table S4. There was no significant main effect of genotype or genotype × gender interaction on any of the demographic, cognitive and psychological variables. However, significant (*P* <0.05) main effect of gender was found in age, years of education, depression and anxiety scores, and harm avoidance of personality. 

**Table 2:** Demographic data for VBM analysis (n = 299) and rsFC analysis (n = 288).

			VBM analysis			rsFC analysis		
			n	Age (years)	Years of education	n	Age (years)	Years of education
COMT	Met carrier		160	22.7 (2.6)	15.4 (2.3)	151	22.7 (2.5)	15.5 (2.2)
	Val/Val		139	22.9 (2.4)	16.0 (2.0)	137	22.9 (2.4)	15.9 (2.0)
	F(P)		299	0.20 (0.65)	3.67 (0.06)	288	0.22 (0.64)	2.46 (0.12)
Gender	Male		141	22.3 (2.6)	15.1 (2.3)	134	22.2 (2.6)	15.1 (2.2)
	Female		158	23.3 (2.3)	16.1 (2.0)	154	23.3 (2.2)	16.2 (1.9)
	F(P)		299	**12.00 (< 0.01)**	**17.18 (< 0.01)**	288	**14.59 (< 0.001)**	**18.22 (< 0.001)**
COMT × gender	Male	Met carrier	79	22.2 (2.7)	14.9 (2.3)	74	22.1 (2.5)	15.0 (2.2)
		Val/Val	62	22.3 (2.6)	15.4 (2.2)	60	22.3 (2.6)	15.3 (2.2)
	Female	Met carrier	81	23.2 (2.4)	16.0 (2.1)	77	23.3 (2.3)	16.0 (2.1)
		Val/Val	77	23.3 (2.2)	16.4 (1.8)	77	23.3 (2.2)	16.4 (1.8)
		F(P)	299	0.11 (0.92)	0.01 (0.96)	288	0.11 (0.92)	0.01 (0.96)

The data are shown as the means (SD).

### GMV differences

**Figure 1:**
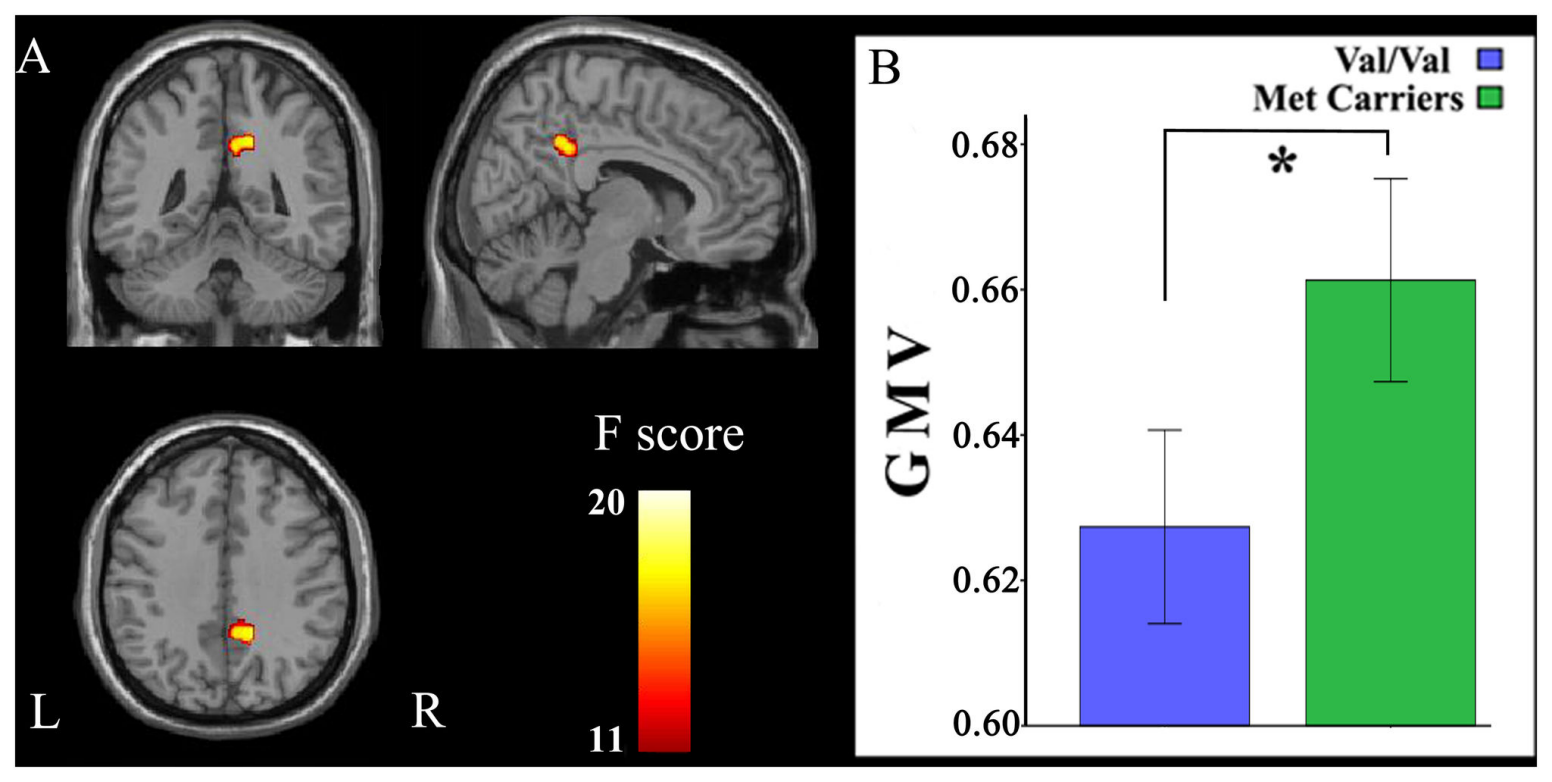
Significant GMV differences between genotypes. Val homozygotes exhibit significantly (*P* < 0.05, corrected) smaller GMV in the right PCC than Met allele carriers. GMV, gray matter volume; L, left; PCC, posterior cingulate cortex; R, right.

**Figure 2:**
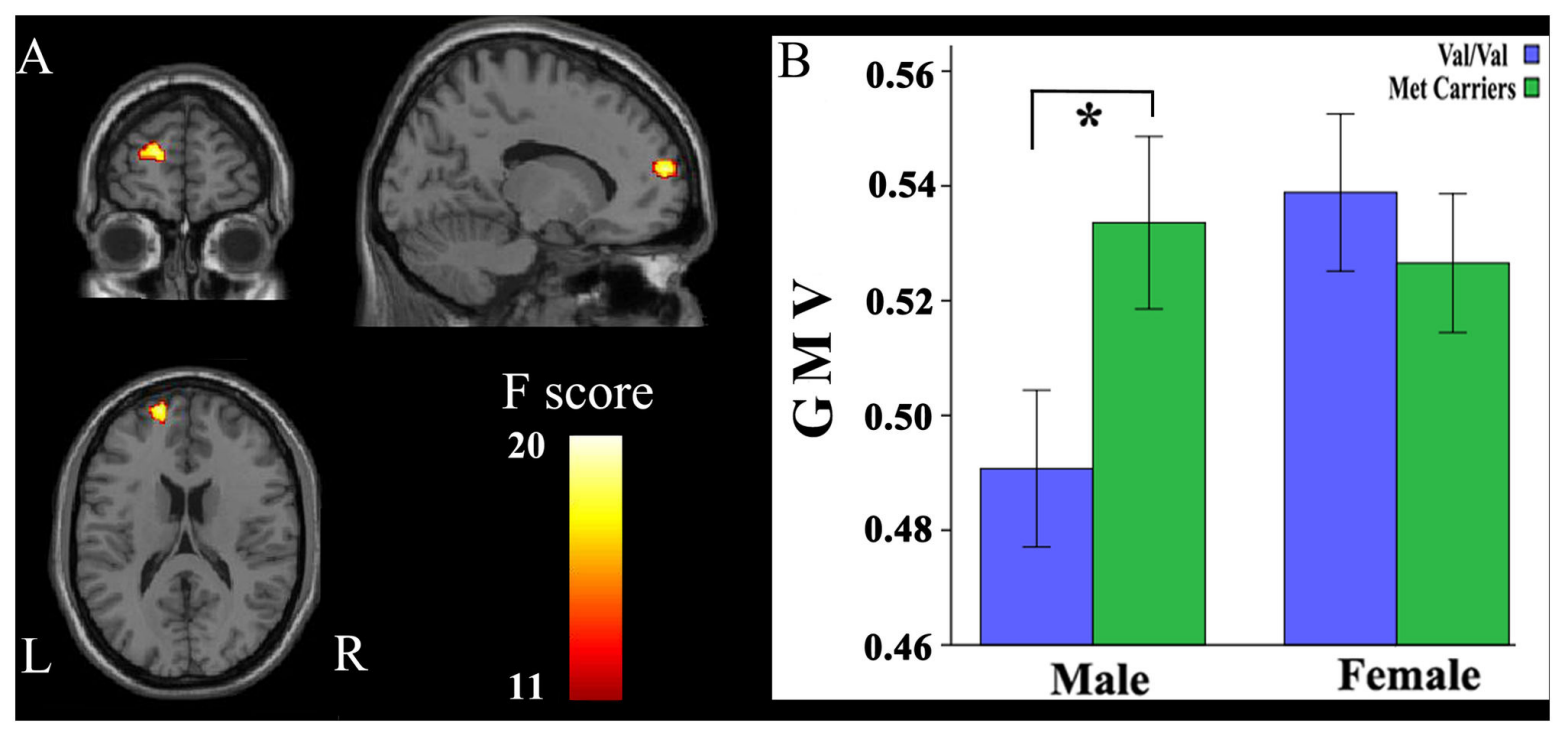
The interaction between genotype and gender in GMV. A significant (*P* < 0.05, corrected) genotype × gender interaction effect is observed in the left mSFG. Only male Val homozygotes exhibit significantly smaller GMV than male Met allele carriers. GMV, gray matter volume; L, left; mSFG, medial superior frontal gyrus; R, right.

The main effects of the GMV differences between genotypes are shown in Figure 1. ANOVA revealed a significant main effect of genotype on GMV in the right posterior cingulate cortex (PCC) (BA 31; peak MNI coordinate: x = 15, y = -46.5, z = 37.5; 233 voxels; peak F = 14.99). *Post-hoc* testing showed that Val homozygotes exhibited significantly smaller GMV in the right PCC than Met allele carriers. The interaction between genotype and gender in GMV is shown in Figure 2. ANOVA demonstrated a significant interaction between genotype and gender in the GMV of the left medial superior frontal gyrus (mSFG) (BA 10; peak MNI coordinate: x = -16.5, y = 60, z = 16.5; 231 voxels; peak F = 17.67). The *post-hoc* comparison showed that only male Val homozygotes exhibited significantly smaller GMV than male Met allele carriers. The main effects of the GMV differences between genders are shown in Figure S1. The gender differences in GMV were found in many brain regions, including the right PCC region that showed a significant main effect of genotype. *Post-hoc* testing showed that male subjects exhibited significantly smaller GMV in the right PCC than female subjects.

### rsFC differences

**Figure 3:**
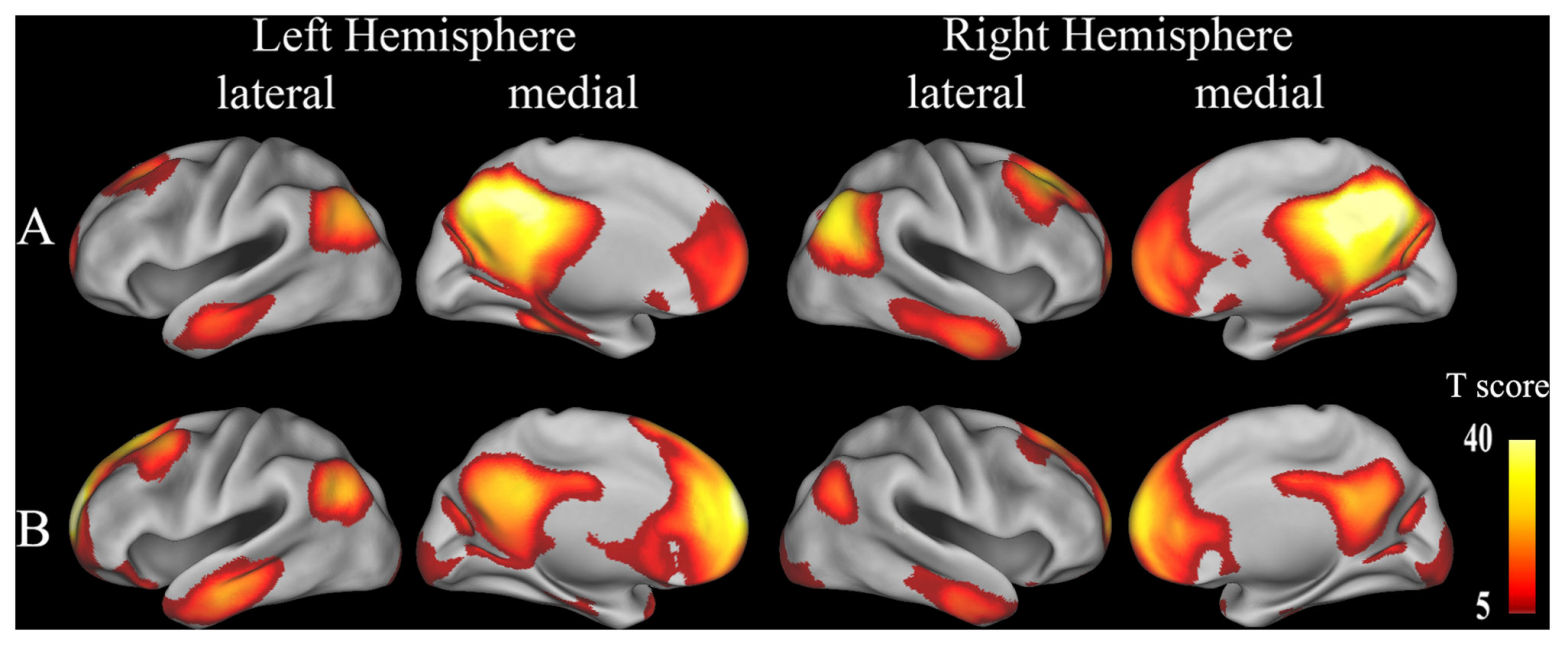
The rsFC maps of the right PCC and left mSFG. One-sample t-test (FWE, *P* < 0.05) reveals that the right PCC is positively correlated with brain regions of the DMN (A). The left mSFG shows a similar rsFC pattern (B), although the right PCC and left mSFG differed in rsFC strengths. DMN, default mode network; FWE, Family Wise Error; L, left; mSFG, medial superior frontal gyrus; PCC, posterior cingulate cortex; R, right; rsFC, resting-state functional connectivity.

**Figure 4:**
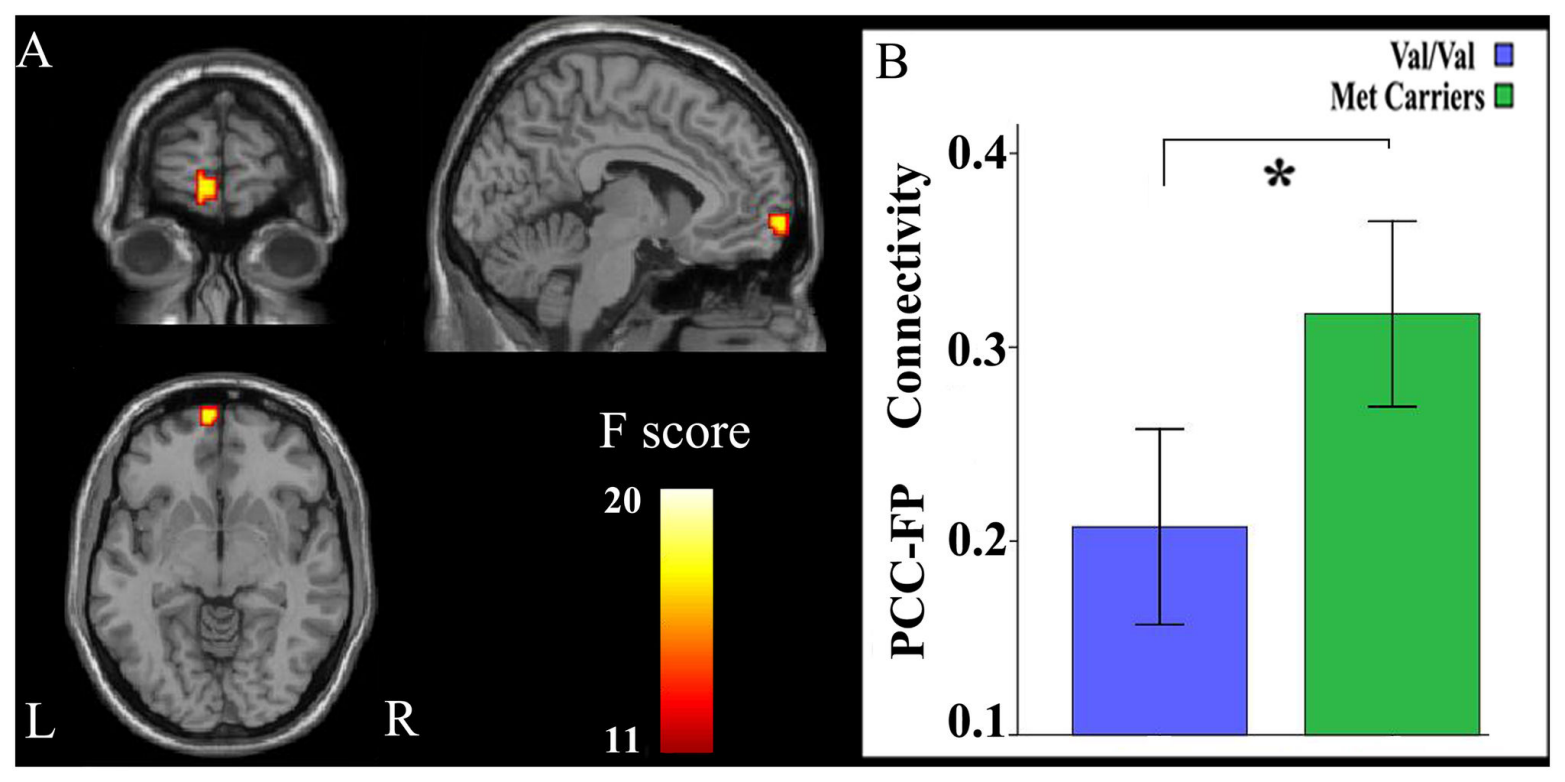
Genotypic differences in the rsFCs of the right PCC. There is only a significant (*P* < 0.05, corrected) main effect of genotype in the rsFC between the right PCC and the left medial FP. Val homozygotes exhibit decreased rsFC when compared with Met allele carriers. FP, frontal pole; L, left; PCC, posterior cingulate cortex; R, right; rsFC, resting-state functional connectivity.

**Figure 5:**
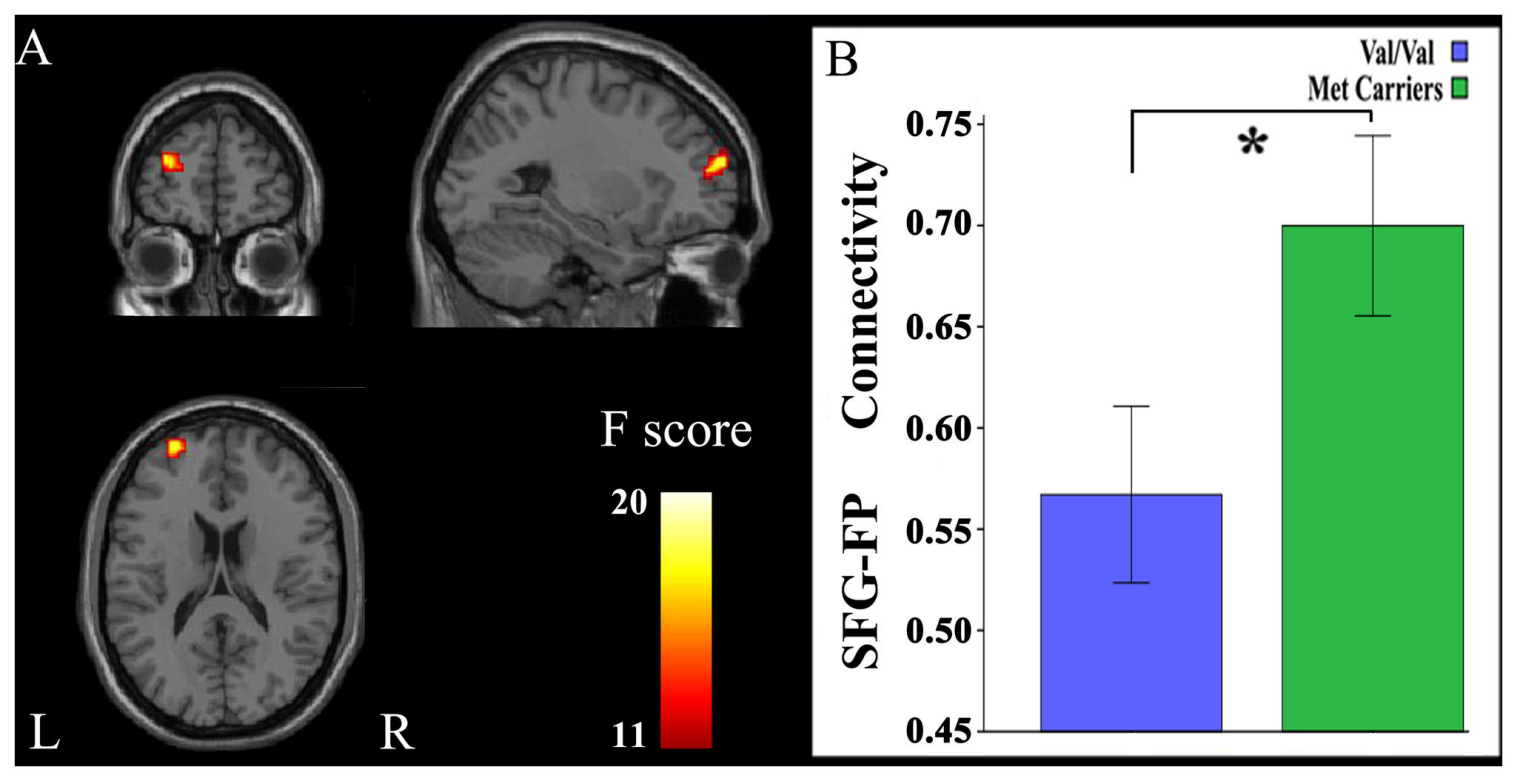
Genotypic differences in the rsFCs of the left mSFG. There is only a significant (*P* < 0.05, corrected) main effect of genotype in the rsFC between the left mSFG and the left FP. Val homozygotes exhibit decreased rsFC when compared with Met allele carriers. FP, frontal pole; L, left; mSFG, medial superior frontal gyrus; R, right; rsFC, resting-state functional connectivity.

For seed regions in the rsFC analysis, we extracted the right PCC because of the significant difference observed in GMV between genotypes, and the left mSFG was extracted because of the significant interaction identified between genotype and gender in GMV. A one-sample t-test (FWE, *P* < 0.05) revealed that these seed regions showed similar rsFC patterns and that they were positively correlated with brain regions of the DMN, including the PCC, precuneus, medial prefrontal cortex, anterior cingulate cortex, lateral parietal cortex, anterior temporal lobe, and SFG, although these regions differed in rsFC strengths (Figure 3). This finding suggests that both the right PCC and the left mSFG are components of the DMN. Voxel-based comparisons of rsFCs were performed using a factorial ANOVA (genotype by gender). When the right PCC was treated as the seed region, there was a significant main effect of genotype on the rsFC between the right PCC and the left medial frontal pole (FP) (BA10; peak MNI coordinate: x = -6, y = 66, z = -6; 24 voxels; peak F = 14.20; *P* < 0.05, corrected) (Figure 4). *Post-hoc* testing showed that Val homozygotes exhibited decreased rsFC compared with Met allele carriers. The main effects of the rsFC differences of the right PCC between genders are shown in Figure S2. The gender differences in rsFCs of the right PCC were found in several brain regions, including the left FP that showed a significant main effect of genotype. *Post-hoc* testing showed that male subjects exhibited significantly weaker rsFC between the right PCC and the left FP than female subjects. Similarly, when the left mSFG was treated as the seed region, there was also a significant main effect of genotype on the rsFC between the seed region and the left FP (BA10; peak MNI coordinate: x = -27, y = 57, z = 18; 23 voxels; peak F = 16.39; *P* < 0.05, corrected) (Figure 5). *Post-hoc* testing showed that Val homozygotes also exhibited decreased rsFC compared with Met allele carriers. However, no significant main effect of gender on the rsFC between the left mSFG and the left FP were found (Figure S3). No significant interactions were observed between genotype and gender in any of the rsFCs studied. After controlling for age, years of education, IQ, depression scores, anxiety levels and personality traits, we found the results of GMV and rsFC analyses were very similar with those without controlling for these factors (Table S5). To test the structural-functional relationship, we repeated the rsFC analysis while controlling for the GMV of the seed region; however, we did not find any significant changes between results with and without correcting for the GMV (Table S6). 

**Figure 6:**
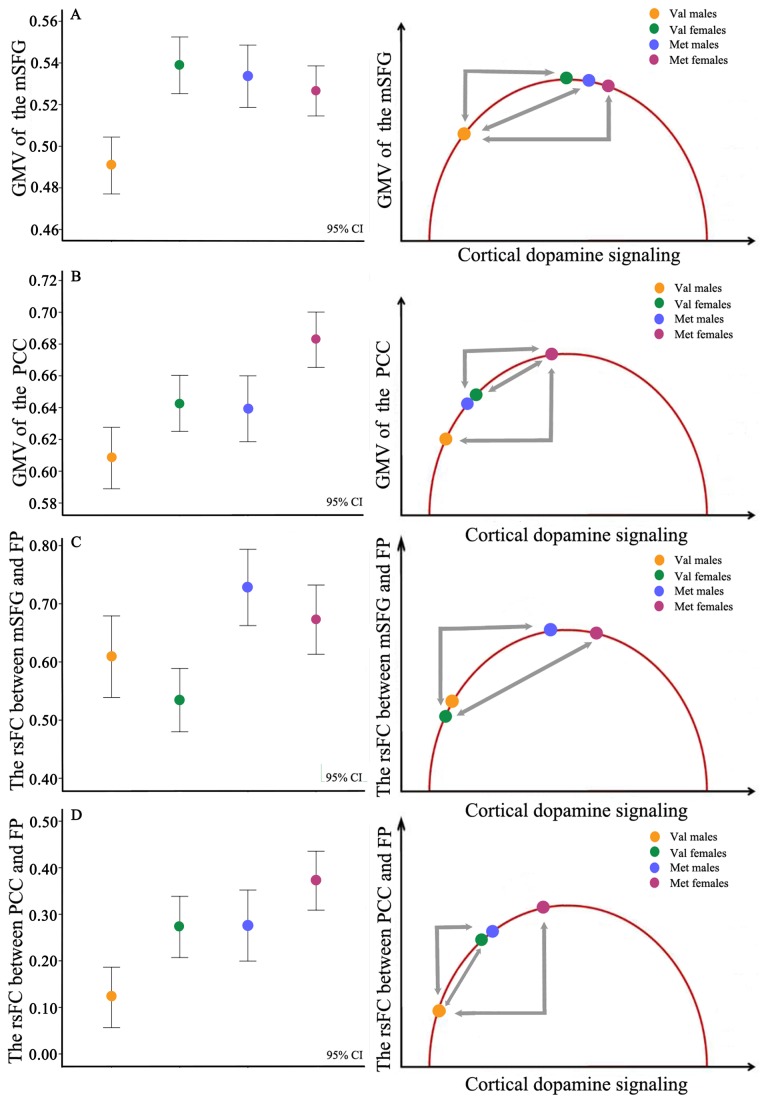
Illustration of the nonlinear curve model explaining the GMV and rsFC differences among different subgroups. Both COMT genotype and gender affect DA availability in the brain. Different DA levels may explain the GMV and rsFC differences that were observed among different subgroups. The left column shows the mean and 95% CI of imaging phenotypes of the four subgroups, whereas the right column shows the location of each imaging phenotype on the curve. Arrows represent significant group differences (*P* < 0.05, corrected). CI, confidence interval; COMT, catechol-O-methyltransferase; DA, dopamine; DMN, default mode network; FP, frontal pole; GMV, gray matter volume; mSFG, medial superior frontal gyrus; PCC, posterior cingulate cortex; rsFC, resting-state functional connectivity.

According the genotype and gender, subjects were divided into four subgroups with different dopamine availability: Val/Val males, Val/Val females, male Met carriers, and female Met carriers. Met-allele carriers have lower COMT activity and higher dopamine availability than Val/Val individuals; and females have higher level of estrogen, lower COMT activity, and higher dopamine availability compared with males. Thus the dopamine signaling is theoretically the lowest in Val/Val males and the highest in female Met carriers. The left column of Figure 6 shows the mean GMV or rsFC of individual subgroup; whereas the right column of Figure 6 fits the mean GMV or rsFC of individual subgroup into the inverted U-shaped model of dopaminergic modulation [2,14,36,84].

## Discussion

Considering the interactions between genotype and gender, we completed a stepwise investigation of COMT Val158Met modulation on GMV and rsFC using a voxel-based analysis of the whole brain in healthy young adults. The VBM analysis revealed decreased GMV in the right PCC of Val/Val individuals compared with that of Met carriers; however, decreased GMV of the left mSFG was observed only in male Val/Val subjects. When using these two brain areas as seed regions, the rsFC analysis revealed that both the right PCC and left mSFG were part of the DMN. Moreover, both seed regions showed decreased rsFCs with different parts of the FP of the DMN in Val/Val individuals than in Met carriers. No significant genotype × gender interaction was found in any of the rsFC studies.

To date, GMV differences between COMT genotypes have been explored in five studies of healthy subjects. Although a recent study failed to find any GMV differences between genotypes [51], most studies have reported GMV differences across genotypes in the temporal cortex [44,48], hippocampus [42,44,48,52] and frontal cortex [42]. Although Honea et al. [44] had found significant GMV differences in the left hippocampal region after multiple comparisons corrected at the whole-brain level, the other three studies found genotype differences in GMV using only a small volume correction [42,52] or a ROI analysis [48]. Even with these liberal statistical methods, several studies have reported no effects of COMT genotype on frontal [85,86] or hippocampal volume [87]. This discrepancy may be caused by differences in demographic characteristics, sample size, environmental background, and statistical methods. In the present study, we recruited a large sample of 299 healthy young Chinese Han subjects, performed GMV analysis at the whole-brain level, and considered both the effect of the genotype, gender and their interactions.

We found that Val/Val males showed smaller GMV in the left mSFG than the other three groups, suggesting that COMT Val158Met effects on prefrontal morphology is gender-dependent. As the key enzyme of DA degradation, COMT may account for more than 60% of the DA degradation in the PFC; it thus plays a unique role in regulating DA levels of the PFC [88] because prefrontal DA transporters are scarce [1]. The Met variant results in a fourfold decrease in enzymatic activity at body temperature [1], resulting that Val/Val individuals have greater COMT activity and lower DA availability in the PFC compared with Met carriers. Additionally, estrogen may inhibit COMT activity; this effect is more prominent in the PFC [89]. Males who have lower level of estrogen may have greater COMT activity and lower DA availability relative to females. Consequently, both the Val/Val and male statuses may result in lowest DA availability in the PFC, which may contribute to the smallest GMV in the left mSFG in the Val/Val male group. 

Using VBM analysis of the whole brain, we found significantly reduced GMV in the PCC of Val/Val individuals when compared with Met carriers. Although no studies have reported structural differences between COMT genotypes, functional differences between genotypes in the PCC have been frequently demonstrated [39,78,90-92]. GMV differences in the PCC between genotypes may also be explained by the different levels of COMT activity and DA availability between the two genotypic groups. Of course, this speculation must be validated in future studies because of the relatively low COMT expression in the PCC compared with that in the PFC. In the present study, we also found a main effect of gender on the GMV of the PCC. Although the underlying mechanism is unclear, the effect of estrogen on the COMT activity and DA availability may be one of the possible candidates. 

In the rsFC analyses, we found significant main effects of genotypes on the rsFCs between the right PCC and the left medial FP and between the left mSFG and the left FP. These findings may be also explained by the different levels of COMT activity and DA availability between the two genotypic groups. However, the significant main effect of gender on the rsFC was only present in the rsFC between the right PCC and the left medial FP, suggesting gender plays a different role in these two rsFCs. Although the effect of estrogen on the COMT activity may partly explain for the gender difference in the rsFC between the right PCC and the left medial FP, this mechanism cannot explain for the lack of gender difference in the rsFC between the left mSFG and the left FP. Thus, other mechanisms may be implicated in gender differences in rsFCs and need to be further studied.

One intriguing finding in this study was that modulatory effects of COMT Val158Met were found in both structural and functional characteristics of the DMN. The DMN is an intrinsic brain system that exhibits higher metabolic activity during non-task states [93], plays an important role in human cognitive functions, and is impaired in several neuropsychiatric diseases [94]. Functional connectivity within the DMN has been recognized as being positively correlated with cognitive performance in task states as well as at rest [95]. Similar to our findings, a previous study revealed that Val homozygotes exhibited decreased PFC and PCC related rsFCs within the DMN [39]. Taken together, these findings highlight a possible neural pathway by which the COMT Val158Met polymorphism may affect cognitive functions via modulation of DMN structure and function. For example, when compared with Met carriers, Val homozygotes exhibited smaller GMV and weaker rsFCs within the DMN; this results in reduced cognitive performance [16,21,22,96]. However, we did not find a direct relationship between the structural and functional changes in the DMN, which may suggest that the modulatory effects of COMT Val158Met on the structural and functional characteristics of the DMN are independent with each other. Future studies should be done to verify the results.

Nonlinear effects of COMT genotype on brain structure and function have been reported in previous studies [10-12,15,44,97]. Although the exact neural mechanisms by which COMT Val158Met affects GMVs of the mSFG and PCC are unclear, potential mechanism is DA level-dependent neurotrophic and neurotoxic effects [44]. The effect of DA levels on neuronal survival and growth has been described as an inverted U-shaped curve, in which the optimal level of extracellular DA can induce the expression of BDNF and facilitate neuronal growth [98]. However, both low and high levels of extracellular DA can impair neuronal integrity and survival [99]. For example, excessive extracellular DA levels in dopamine transporter knockout mice can reduce BDNF gene expression in the frontal cortex [100], whereas reduced DA signaling in D1 receptor mutant mice can impair the normal expression of DA-mediated behavioral responses by affecting the neurochemical architecture of the striatum [101]. Pharmacological studies in both animals [102] and humans [14,103-106] have indicated that relatively poor cognitive performance in individuals with lower DA levels tended to improve after dopamimetic agent stimulation, whereas performance of individuals with DA levels near or at the top of the inverted U-shaped curve showed no improvement or deterioration with these agents. Our findings may partly support the inverted U-shaped model in that Val/Val males who have the greatest COMT activity and the lowest DA availability exhibited the smallest GMV and the weakest rsFC among the four groups. However, no indication of the aversive effect of high DA availability on GMV or rsFC (the downside downslope of the inverted-U) (Figure 6) may weaken the inverted U-shaped hypothesis. 

It should be noted that different preprocessing methods had been used in previous rsFC studies. In the present study, we first used a temporal filter, and then remove the confounding factors (such as head motion) using a multiple regression according to several previous studies [82,107-109]. It has been criticized that this preprocessing method may reduce the effectiveness of removing the motion artifact using the regression method. To clarify the issue, we adopted an alternative preprocessing method, that is, we first used a multiple regression to remove motion parameters and other confounding factors, and then we used a temporal filter to reduce the effects of low-frequency drifts and high-frequency noise. We repeated the rsFC analysis on the preprocessed fMRI data with a different procedure and found that the new results (Figure S4 and Figure S5) were the same as the original results (Figure 4 and Figure 5) using the same statistical threshold (*P* < 0.05, corrected). These results suggest that the order of the preprocessing steps did not significantly influence our findings. In the present study, we used two methods for multiple comparison correction. The FWE is a stronger correction method than the Monte Carlo simulation. The selection of different correction methods depends on the effective sizes. The one-sample t-test is to identify brain regions exhibiting significant positive correlations with the seed region. The effective size is relative large and then a stricter FWE correction was used in this situation. In contrast, the effective size of the genotypic differences between healthy adults is expected to be small. Therefore, we used a relatively loose threshold to avoid missing the subtle differences between groups. However, the intergroup differences cannot survive after the FWE correction for multiple comparisons. The lack of significant intergroup differences after a stricter FWE correction suggests that these findings should be validated in future studies.

In summary, this study used a relatively large sample size of healthy young adults and a whole brain analyzing method. We found that the COMT Val158Met polymorphism modulates anatomical morphology and related rsFCs within the DMN, indicating a potential neural pathway by which this polymorphism may affect cognitive function. Meanwhile, we found a genotype × gender interaction in the prefrontal GMV but not in the GMV of the PCC and the rsFCs within the DMN. The mechanisms of these findings need to be further investigated.

## Supporting Information

Figure S1 **Brain regions with significant GMV differences affected by genders (P < 0.05, corrected).** The GMV of mSFG and PCC are effected by gender. GMV, gray matter volume; mSFG, medial superior frontal gyrus; L, left; PCC, posterior cingulate cortex; R, right.
(DOC)

Figure S2 **Brain regions with significant gender differences in rsFCs of the right PCC (P < 0.05, corrected).** There is a significant main effect of gender on several brain regions, including the left FP that showed a significant main effect of genotype. FP, frontal pole; L, left; PCC, posterior cingulate cortex; R, right; rsFC, resting-state functional connectivity.
(DOC)

Figure S3 **Brain regions with gender differences in the rsFCs of the left mSFG (P < 0.05, corrected).** L, left; mSFG, medial superior frontal gyrus; R, right; rsFC, resting-state functional connectivity.
(DOC)

Figure S4 **Genotypic differences in the rsFCs of the right PCC.** There is only a significant (P < 0.05, corrected) main effect of genotype in the rsFC between the right PCC and the left medial FP. Val homozygotes exhibit decreased rsFC when compared with Met allele carriers. FP, frontal pole; L, left; PCC, posterior cingulate cortex; R, right; rsFC, resting-state functional connectivity.
(DOC)

Figure S5 **Genotypic differences in the rsFCs of the left mSFG.** There is only a significant (P < 0.05, corrected) main effect of genotype in the rsFC between the left mSFG and the left FP. Val homozygotes exhibit decreased rsFC when compared with Met allele carriers. FP, frontal pole; L, left; mSFG, medial superior frontal gyrus; R, right; rsFC, resting-state functional connectivity.
(DOC)

Table S1 **Assessment of head motion in fMRI data using framewise displacement (FD) measure.**
(DOC)

Table S2 **Demographic data and IQ scores of subjects (n = 297).**
(DOC)

Table S3 **Demographic data and depression scores and anxiety values of subjects (n = 279).**
(DOC)

Table S4 **Demographic data and personality traits of subjects (n = 292).**
(DOC)

Table S5 **Statistical results before (out of the brackets) and after (in the brackets) removing demographic data and behavioral scales.**
(DOC)

Table S6 **Statistical results before (out of the brackets) and after (in the brackets) removing the GMV of seed region.**
(DOC)
