# Supplementary material for: Catechol-O-Methyltransferase Val158Met Polymorphism Modulates Gray Matter Volume and Functional Connectivity of the Default Mode Network
Source: PLoS One. 2013 Oct 16;8(10):e78697. doi: 10.1371/journal.pone.0078697 (PMC3797700; doi:10.1371/journal.pone.0078697)
Supplement: Figure S1 — Brain regions with significant GMV differences affected by genders (P < 0.05, corrected). The GMV of mSFG and PCC are effected by gender. GMV, gray matter volume; mSFG, medial superior frontal gyrus; L, left; PCC, posterior cingulate cortex; R, right. (DOC) [file pone.0078697.s001.doc]

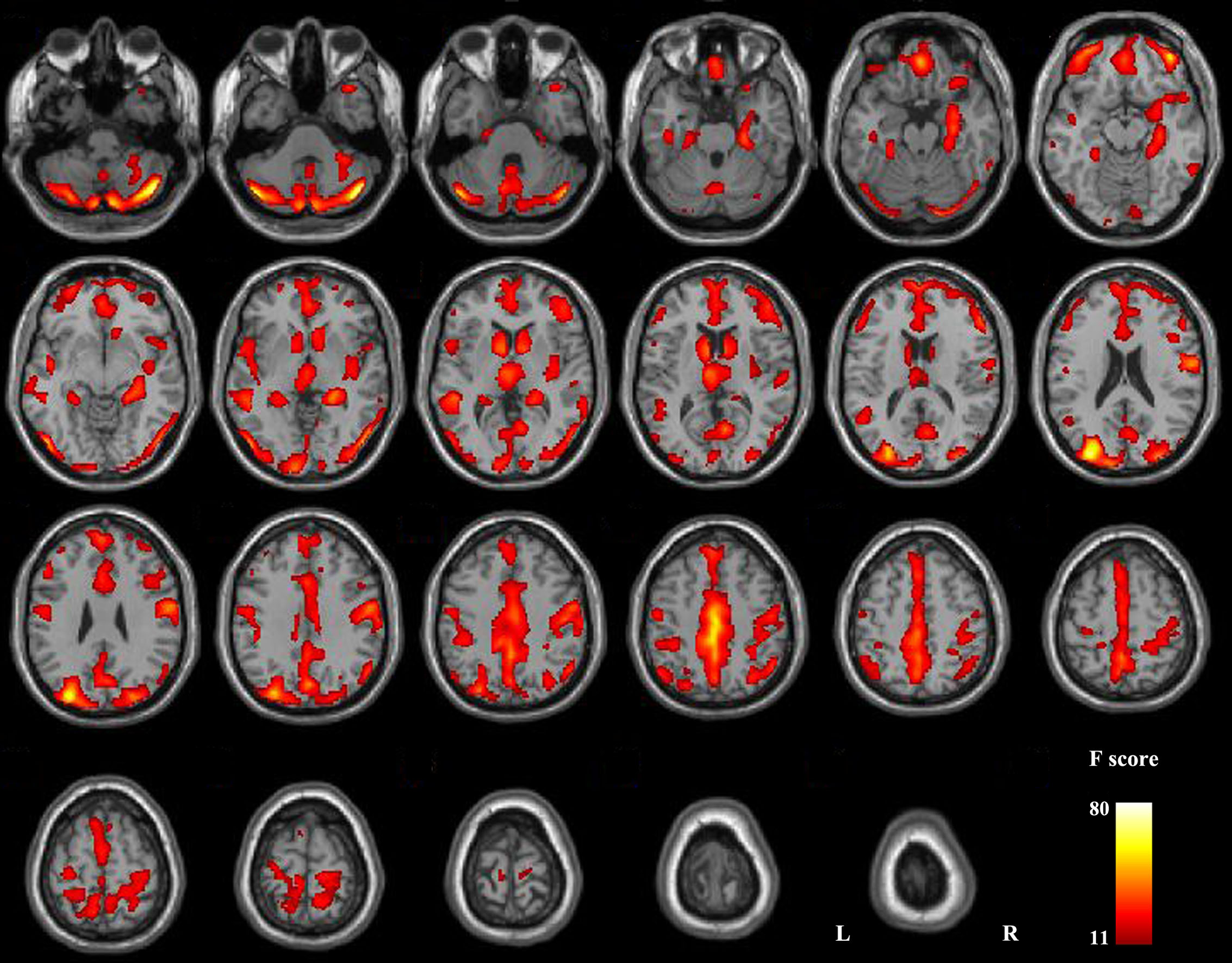


**Figure S1**. Brain regions with significant GMV differences affected by genders (*P* < 0.05, corrected). The GMV of mSFG and PCC are effected by gender. GMV, gray matter volume; mSFG, medial superior frontal gyrus; L, left; PCC, posterior cingulate cortex; R, right.
