# Supplementary material for: Catechol-O-Methyltransferase Val158Met Polymorphism Modulates Gray Matter Volume and Functional Connectivity of the Default Mode Network
Source: PLoS One. 2013 Oct 16;8(10):e78697. doi: 10.1371/journal.pone.0078697 (PMC3797700; doi:10.1371/journal.pone.0078697)
Supplement: Figure S2 — Brain regions with significant gender differences in rsFCs of the right PCC (P < 0.05, corrected). There is a significant main effect of gender on several brain regions, including the left FP that showed a significant main effect of genotype. FP, frontal pole; L, left; PCC, posterior cingulate cortex; R, right; rsFC, resting-state functional connectivity. (DOC) [file pone.0078697.s002.doc]

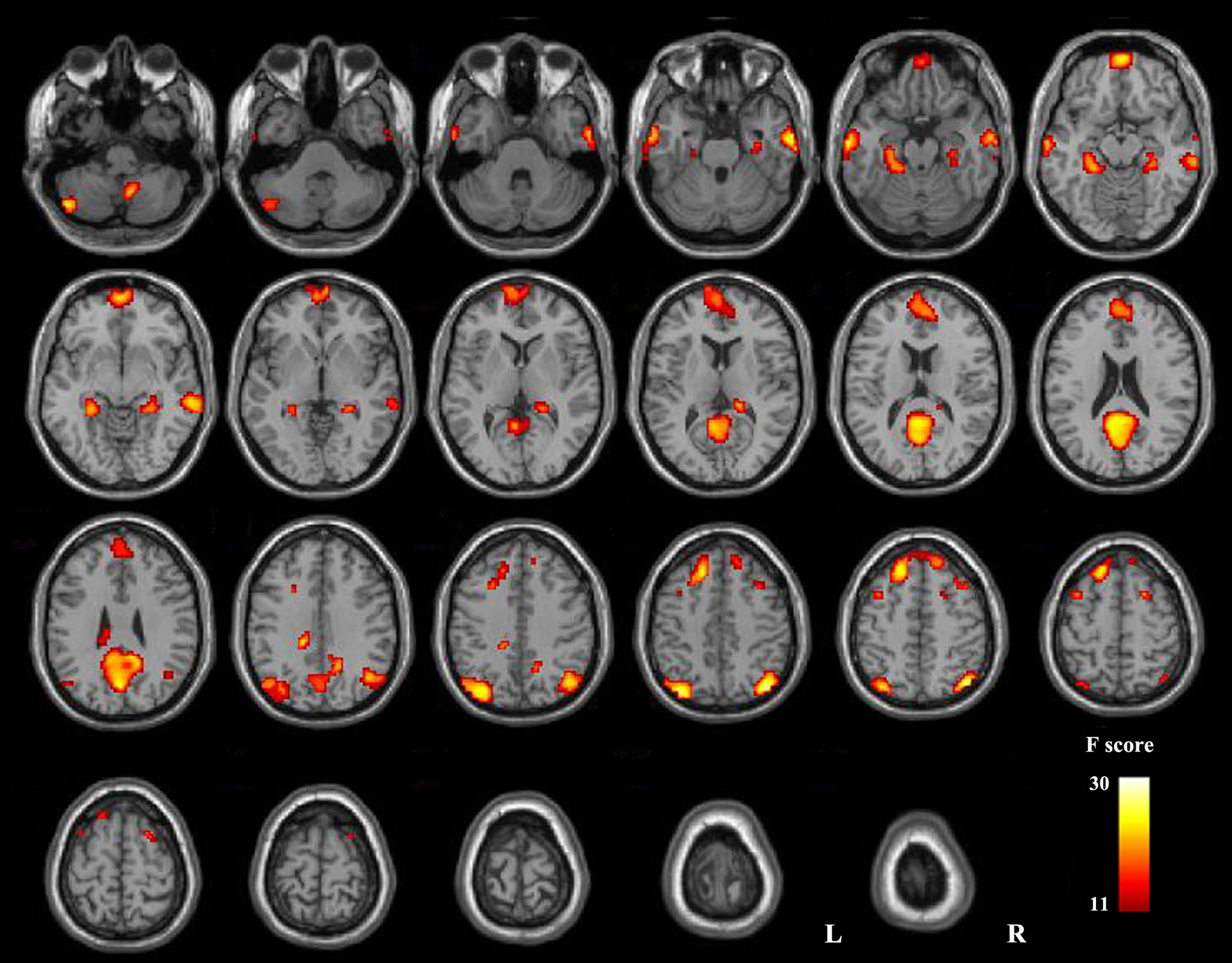


**Figure S2**. Brain regions with significant gender differences in rsFCs of the right PCC (*P* < 0.05, corrected). There is a significant main effect of gender on several brain regions, including the left FP that showed a significant main effect of genotype. FP, frontal pole; L, left; PCC, posterior cingulate cortex; R, right; rsFC, resting-state functional connectivity.
