# Supplementary material for: Catechol-O-Methyltransferase Val158Met Polymorphism Modulates Gray Matter Volume and Functional Connectivity of the Default Mode Network
Source: PLoS One. 2013 Oct 16;8(10):e78697. doi: 10.1371/journal.pone.0078697 (PMC3797700; doi:10.1371/journal.pone.0078697)
Supplement: Figure S3 — Brain regions with gender differences in the rsFCs of the left mSFG (P < 0.05, corrected). L, left; mSFG, medial superior frontal gyrus; R, right; rsFC, resting-state functional connectivity. (DOC) [file pone.0078697.s003.doc]

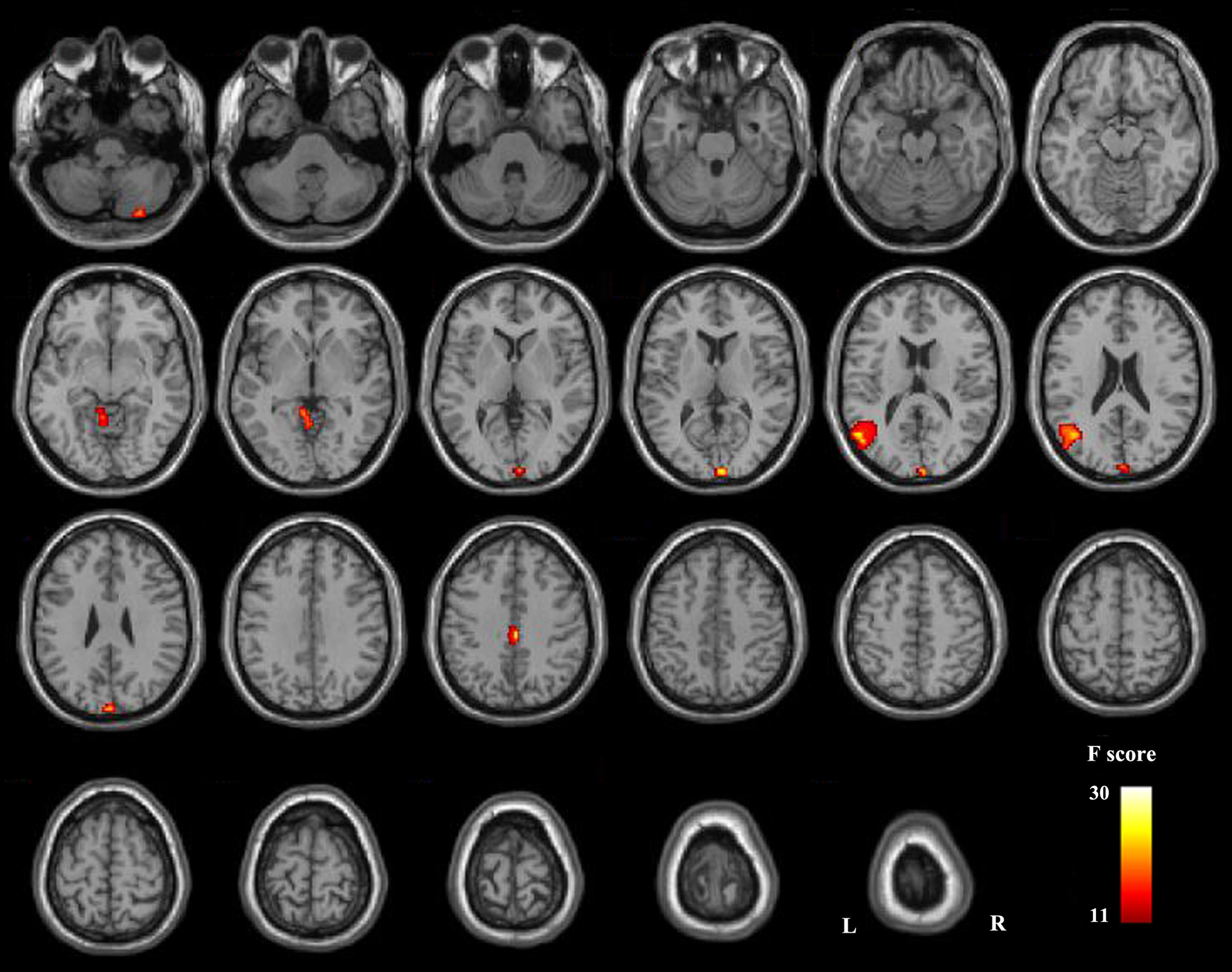


**Figure S3**. Brain regions with gender differences in the rsFCs of the left mSFG (*P* < 0.05, corrected). L, left; mSFG, medial superior frontal gyrus; R, right; rsFC, resting-state functional connectivity.
