# Supplementary material for: Catechol-O-Methyltransferase Val158Met Polymorphism Modulates Gray Matter Volume and Functional Connectivity of the Default Mode Network
Source: PLoS One. 2013 Oct 16;8(10):e78697. doi: 10.1371/journal.pone.0078697 (PMC3797700; doi:10.1371/journal.pone.0078697)
Supplement: Figure S5 — Genotypic differences in the rsFCs of the left mSFG. There is only a significant (P < 0.05, corrected) main effect of genotype in the rsFC between the left mSFG and the left FP. Val homozygotes exhibit decreased rsFC when compared with Met allele carriers. FP, frontal pole; L, left; mSFG, medial superior frontal gyrus; R, right; rsFC, resting-state functional connectivity. (DOC) [file pone.0078697.s005.doc]

**
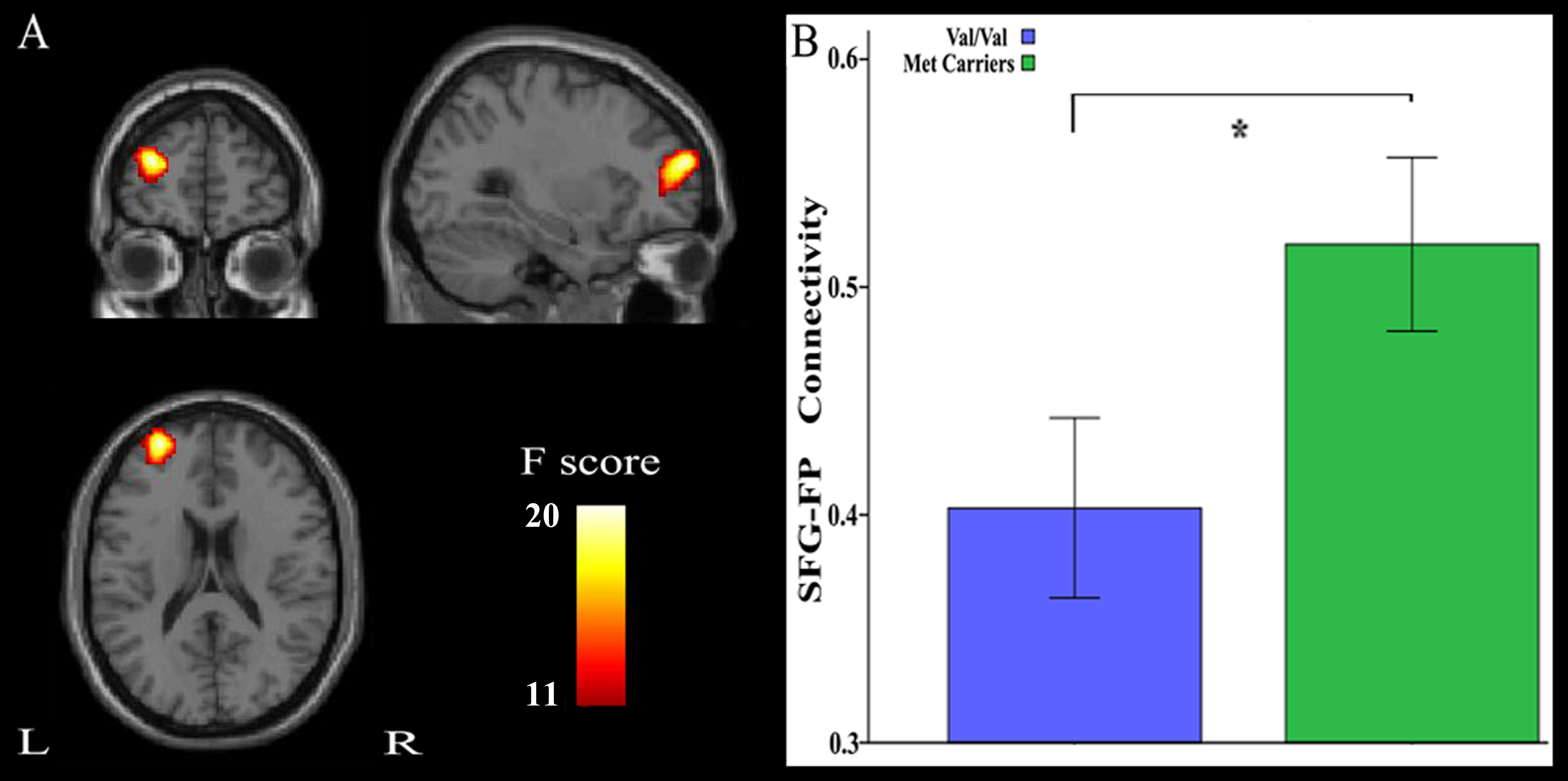
**

**Figure S5.** Genotypic differences in the rsFCs of the left mSFG. There is only a significant (*P* < 0.05, corrected) main effect of genotype in the rsFC between the left mSFG and the left FP. Val homozygotes exhibit decreased rsFC when compared with Met allele carriers. FP, frontal pole; L, left; mSFG, medial superior frontal gyrus; R, right; rsFC, resting-state functional connectivity.
