# Supplementary material for: Catechol-O-Methyltransferase Val158Met Polymorphism Modulates Gray Matter Volume and Functional Connectivity of the Default Mode Network
Source: PLoS One. 2013 Oct 16;8(10):e78697. doi: 10.1371/journal.pone.0078697 (PMC3797700; doi:10.1371/journal.pone.0078697)
Supplement: Table S1 — Assessment of head motion in fMRI data using framewise displacement (FD) measure. (DOC) [file pone.0078697.s006.doc]

Table S1. Assessment of head motion in fMRI data using framewise displacement (FD) measure.

|  | COMT | | | COMT × gender | | | | |
| --- | --- | --- | --- | --- | --- | --- | --- | --- |
|  | Met carrier | Val/Val | F(*P*) | Male | | Female | | F(*P*) |
|  |  |  |  | Met carrier | Val/Val | Met carrier | Val/Val |  |
| n | 151 | 137 | 288 | 74 | 60 | 77 | 77 | 288 |
| FD | 0.092 (0.054) | 0.083 (0.041) | 1.675 (0.197) | 0.099 (0.066) | 0.093 (0.050) | 0.084 (0.038) | 0.076 (0.030) | 0.018 (0.892) |

The data are shown as the means (SD).
