# Supplementary material for: Catechol-O-Methyltransferase Val158Met Polymorphism Modulates Gray Matter Volume and Functional Connectivity of the Default Mode Network
Source: PLoS One. 2013 Oct 16;8(10):e78697. doi: 10.1371/journal.pone.0078697 (PMC3797700; doi:10.1371/journal.pone.0078697)
Supplement: Table S2 — Demographic data and IQ scores of subjects (n = 297). (DOC) [file pone.0078697.s007.doc]

Table S2. Demographic data and IQ scores of subjects (n = 297).

|  | | | n | Age (years) | Years of education | VIQ | PIQ | FSIQ |
| --- | --- | --- | --- | --- | --- | --- | --- | --- |
| COMT | Met carrier | | 158 | 22.7 (2.6) | 15.4 (2.3) | 116.7 (9.6) | 112.0 (10.6) | 116.1 (8.9) |
|  | Val/Val | | 139 | 22.9 (2.4) | 15.9 (2.0) | 118.5 (9.0) | 113.6 (9.4) | 118.1 (8.0) |
|  | F(*P*) | | 297 | 0.37 (0.55) | 3.65 (0.06) | 2.35 (0.13) | 1.72 (0.19) | 3.50 (0.06) |
| Gender | Male | | 140 | 22.2 (2.6) | 15.1 (2.3) | 116.6 (9.8) | 113.1 (10.7) | 116.5 (9.3) |
|  | Female | | 157 | 23.2 (2.3) | 16.2 (2.0) | 118.4 (8.8) | 112.4 (9.5) | 117.4 (7.9) |
|  | F(*P*) | | 297 | **11.85 (< 0.001)** | **17.43 (< 0.001)** | 2.42 (0.12) | 0.30 (0.59) | 0.85 (0.36) |
| COMT × gender | Male | Met carrier | 78 | 22.1 (2.7) | 14.9 (2.4) | 116.0 (10.3) | 113.3 (11.3) | 116.3 (9.7) |
|  |  | Val/Val | 62 | 22.3 (2.6) | 15.4 (2.2) | 117.3 (9.2) | 113.0 (10.0) | 116.8 (8.7) |
|  | Female | Met carrier | 80 | 23.2 (2.4) | 15.9 (2.1) | 117.4 (8.8) | 110.8 (9.7) | 116.0 (8.2) |
|  |  | Val/Val | 77 | 23.3 (2.2) | 16.4 (1.8) | 119.4 (8.7) | 114.2 (9.0) | 119.0 (7.3) |
|  |  | F(*P*) | 297 | 0.01 (0.93) | 0.01 (0.91) | 0.11 (0.74) | 2.59 (0.11) | 1.68 (0.20) |

The data are shown as the means (SD). FSIQ, full scale intelligence quotient; PIQ, performance intelligence quotient; VIQ, verbal intelligence quotient.
