# Supplementary material for: Catechol-O-Methyltransferase Val158Met Polymorphism Modulates Gray Matter Volume and Functional Connectivity of the Default Mode Network
Source: PLoS One. 2013 Oct 16;8(10):e78697. doi: 10.1371/journal.pone.0078697 (PMC3797700; doi:10.1371/journal.pone.0078697)
Supplement: Table S3 — Demographic data and depression scores and anxiety values of subjects (n = 279). (DOC) [file pone.0078697.s008.doc]

Table S3. Demographic data and depression scores and anxiety values of subjects (n = 279).

|  | | | n | Age (years) | Years of education | SAS | BDI |
| --- | --- | --- | --- | --- | --- | --- | --- |
| COMT | Met carrier | | 149 | 22.7 (2.5) | 15.5 (2.3) | 30.0 (6.0) | 7.5 (6.1) |
|  | Val/Val | | 130 | 22.9 (2.4) | 16.0 (2.0) | 30.4 (6.0) | 7.5 (6.7) |
|  | F(*P*) | | 279 | 0.29 (0.59) | 3.67 (0.06) | 0.34 (0.56) | 0.00 (0.98) |
| Gender | Male | | 129 | 22.4 (2.6) | 15.2 (2.3) | 31.0 (6.5) | 8.8 (7.0) |
|  | Female | | 150 | 23.2 (2.3) | 16.1 (1.9) | 29.5 (5.4) | 6.4 (5.6) |
|  | F(*P*) | | 279 | **6.81 (0.01)** | **11.14 (< 0.001)** | **4.31 (0.04)** | **9.63 (< 0.001)** |
| COMT × gender | Male | Met carrier | 71 | 22.3 (2.7) | 15.0 (2.4) | 30.9 (6.7) | 8.9 (6.6) |
|  |  | Val/Val | 58 | 22.5 (2.6) | 15.5 (2.2) | 31.1 (6.2) | 8.7 (7.4) |
|  | Female | Met carrier | 78 | 23.1 (2.4) | 15.9 (2.0) | 29.2 (5.0) | 6.4 (5.4) |
|  |  | Val/Val | 72 | 23.2 (2.2) | 16.4 (1.8) | 29.9 (5.8) | 6.5 (5.8) |
|  |  | F(*P*) | 279 | <0.001 (0.99) | 0.01 (0.95) | 0.09 (0.77) | 0.04 (0.84) |

The data are shown as the means (SD). BDI, Beck depression inventory; SAS, Self-Rating Anxiety Scale.
