# Supplementary material for: Catechol-O-Methyltransferase Val158Met Polymorphism Modulates Gray Matter Volume and Functional Connectivity of the Default Mode Network
Source: PLoS One. 2013 Oct 16;8(10):e78697. doi: 10.1371/journal.pone.0078697 (PMC3797700; doi:10.1371/journal.pone.0078697)
Supplement: Table S4 — Demographic data and personality traits of subjects (n = 292). (DOC) [file pone.0078697.s009.doc]

Table S4. Demographic data and personality traits of subjects (n = 292).

|  | | | n | Age (years) | Years of education | NS | HA | RD |
| --- | --- | --- | --- | --- | --- | --- | --- | --- |
| COMT | Met carrier | | 156 | 22.7 (2.6) | 15.4 (2.3) | 14.5 (5.0) | 14.0 (6.2) | 19.2 (3.5) |
|  | Val/Val | | 136 | 22.9 (2.4) | 15.9 (2.1) | 14.2 (5.2) | 15.3 (6.5) | 19.0 (3.3) |
|  | F(*P*) | | 292 | 0.06 (0.81) | 3.06 (0.08) | 0.35 (0.56) | 2.70 (0.10) | 0.11 (0.74) |
| Gender | Male | | 137 | 22.3 (2.6) | 15.1 (2.3) | 14.5 (4.7) | 13.4 (6.3) | 19.2 (3.3) |
|  | Female | | 155 | 23.3 (2.3) | 16.1 (2.0) | 14.2 (5.4) | 15.7 (6.3) | 19.0 (3.5) |
|  | F(*P*) | | 292 | **12.00 (<0.001)** | **16.08 (<0.001)** | 0.25 (0.61) | **9.60 (<0.001)** | 0.10 (0.75) |
| COMT×gender | Male | Met carrier | 77 | 22.2 (2.7) | 14.9 (2.4) | 14.8 (4.7) | 13.2 (6.6) | 19.2 (3.6) |
|  |  | Val/Val | 60 | 22.3 (2.5) | 15.4 (2.2) | 14.2 (4.7) | 13.6 (5.9) | 19.1 (3.0) |
|  | Female | Met carrier | 79 | 23.2 (2.4) | 15.9 (2.1) | 14.2 (5.3) | 14.7 (5.8) | 19.1 (3.5) |
|  |  | Val/Val | 76 | 23.3 (2.2) | 16.4 (1.8) | 14.2 (5.6) | 16.7 (6.7) | 19.0 (3.6) |
|  |  | F(*P*) | 292 | <0.001 (0.99) | <0.001 (0.96) | 0.27 (0.61) | 1.12 (0.29) | <0.001 (0.97) |

The data are shown as the means (SD). HA, harm avoiding; NS, novelty seeking; RD, reward depending.
