# Supplementary material for: Catechol-O-Methyltransferase Val158Met Polymorphism Modulates Gray Matter Volume and Functional Connectivity of the Default Mode Network
Source: PLoS One. 2013 Oct 16;8(10):e78697. doi: 10.1371/journal.pone.0078697 (PMC3797700; doi:10.1371/journal.pone.0078697)
Supplement: Table S5 — Statistical results before (out of the brackets) and after (in the brackets) removing demographic data and behavioral scales. (DOC) [file pone.0078697.s010.doc]

Table S5. Statistical results before (out of the brackets) and after (in the brackets) removing demographic data and behavioral scales.

| **Dependent variable** | **Effects** | **F score** | ***P*** |
| --- | --- | --- | --- |
| GMV of PCC | COMT | **11.37 (11.15)** | **0.001 (0.001)** |
|  | Gender | **16.21 (13.31)** | **<0.001 (<0.001)** |
|  | COMT ×Gender | 0.38 (0.61) | 0.54 (0.44) |
| GMV of SFG | COMT | 7.44 (9.26) | 0.007 (0.003) |
|  | Gender | 6.72 (4.88) | 0.01 (0.03) |
|  | COMT ×Gender | **12.63 (11.27)** | **<0.001 (0.001)** |
| PCC-FP connectivety | COMT | **17.92 (15.37)** | **<0.001 (<0.001)** |
|  | Gender | **12.79 (8.50)** | **<0.001 (0.004)** |
|  | COMT ×Gender | 0.11 (0.22) | 0.75 (0.64) |
| SFG-FP connectivety | COMT | **14.17 (16.21)** | **<0.001 (<0.001)** |
|  | Gender | 1.75 (2.40) | 0.19 (0.12) |
|  | COMT ×Gender | 0.01 (<0.01) | 0.93 (0.98) |
