# Supplementary material for: Catechol-O-Methyltransferase Val158Met Polymorphism Modulates Gray Matter Volume and Functional Connectivity of the Default Mode Network
Source: PLoS One. 2013 Oct 16;8(10):e78697. doi: 10.1371/journal.pone.0078697 (PMC3797700; doi:10.1371/journal.pone.0078697)
Supplement: Table S6 — Statistical results before (out of the brackets) and after (in the brackets) removing the GMV of seed region. (DOC) [file pone.0078697.s011.doc]

Table S6. Statistical results before (out of the brackets) and after (in the brackets) removing the GMV of seed region.

| **Dependent variable** | **Effects** | **F score** | ***P*** |
| --- | --- | --- | --- |
| PCC-FP connectivety | COMT | **14.06 (13.63)** | **<0.001 (<0.001)** |
|  | Gender | **13.78 (13.26)** | **<0.001 (<0.001)** |
|  | COMT ×Gender | 0.34 (0.33) | 0.56 (0.56) |
| SFG-FP connectivety | COMT | **18.68 (22.36)** | **<0.001 (<0.001)** |
|  | Gender | 2.51 (1.28) | 0.11 (0.26) |
|  | COMT ×Gender | 0.04 (0.73) | 0.85 (0.39) |
